# Supplementary material for: Paracrine Regulation of Alveolar Epithelial Damage and Repair Responses by Human Lung-Resident Mesenchymal Stromal Cells
Source: Cells. 2021 Oct 23;10(11):2860. doi: 10.3390/cells10112860 (PMC8616441; doi:10.3390/cells10112860)
Supplement: Supplementary file 1 [file cells-10-02860-s001.zip › cells-1421578-supplementary.pdf]

Distal lung tissue cytospin analysis from

Within 1-2 after isolation of unfractionated cell suspensions from distal lung tissue, cytospin slides were prepared from for immuno-histochemical staining. Cytospin slides were stained for using primary antibodies stromal cell marker CD90 (Biologend) or CD29 (Immunotools), endothelial cell marker CD31 (Immunotools) and macrophage marker CD68 (Dako). Slides were blocked with 0.075% H_2O_2 for 30 min at room temperature, incubated with 1:200 primary antibody or isotype control in PBS/1%BSA/0.1%Triton overnight at 4°C, washed, incubated with the secondary antibody (1:200 Rabbit anti-Mouse-peroxidase (Sigma-Aldrich) in 1xPBS/1%BSA/0.1%Triton) for 30 min. Peroxidase was catalyzed using aminoethyl carbazole (AEC) in 50 mM acetate buffer substrate and Hematoxylin counterstaining of the nuclei. Cells were visualized at 10x with bright field microscopy.

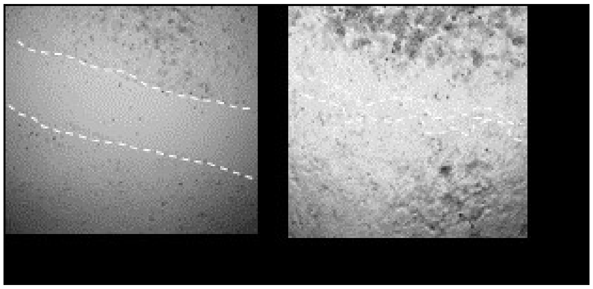

Figure S1. Scratch wounding of NCI-H441 cells. Cells were seeded in duplicates in ECIS arrays and grown to confluence for 48 hours. Cells were wounded by scratching. A representative light microscopy image of scratching of the A549 monolayer and its recovery.

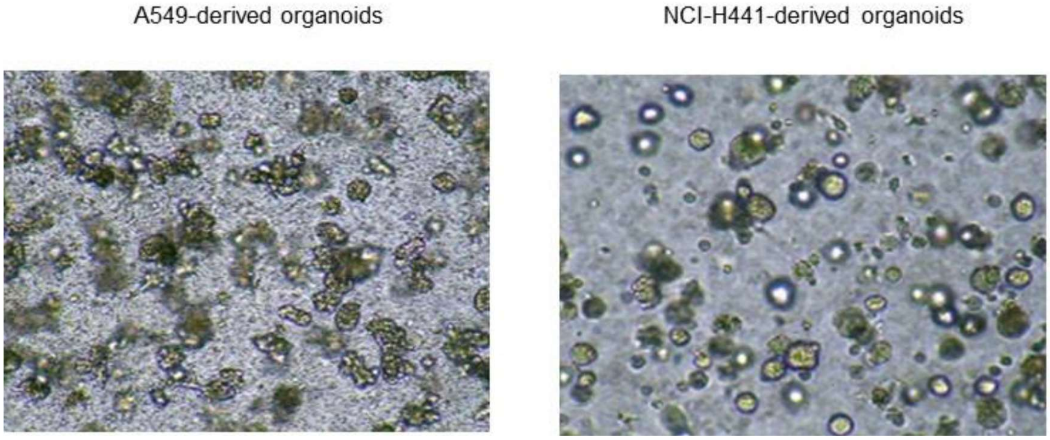

Figure S2. Comparison of organoid formation by A549 and NCI-H441 epithelial cells. Epithelial cells were seeded together with mitomycin-treated MRC-5 cells into 100 μl growth factor-reduced 1:1 diluted Matrigel onto inserts of a transwell and cultured for 14 days.

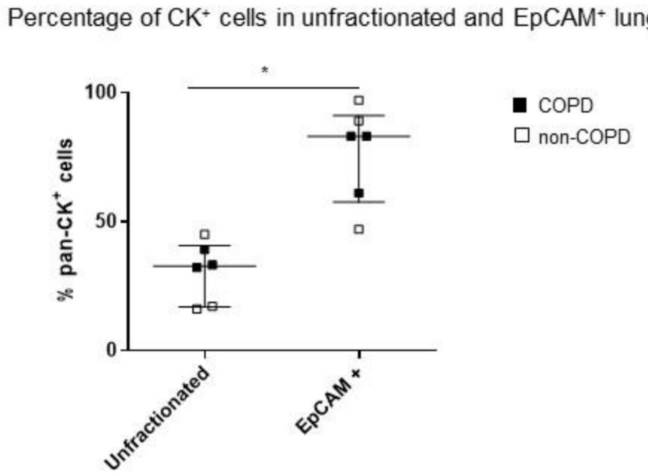

Figure S3. Percentage of epithelial cells in the different cellular fractions used for organoid assays. EpCAM+ or unfractionated cells suspensions were obtained by enzymatic digestion from human distal lung tissue. Cytospins were prepared from unfractionated suspensions and EpCAM+ isolations from 3 control and 3 COPD lungs and stained for pan-cytokeratin as epithelial marker, Medians are indicated. Positive cells were quantified by counting of the complete cytospins by 2 independent observers and expressed as percentage. Medians are indicated. *=p<0.05 between indicated as determined by the Mann Whitney test.

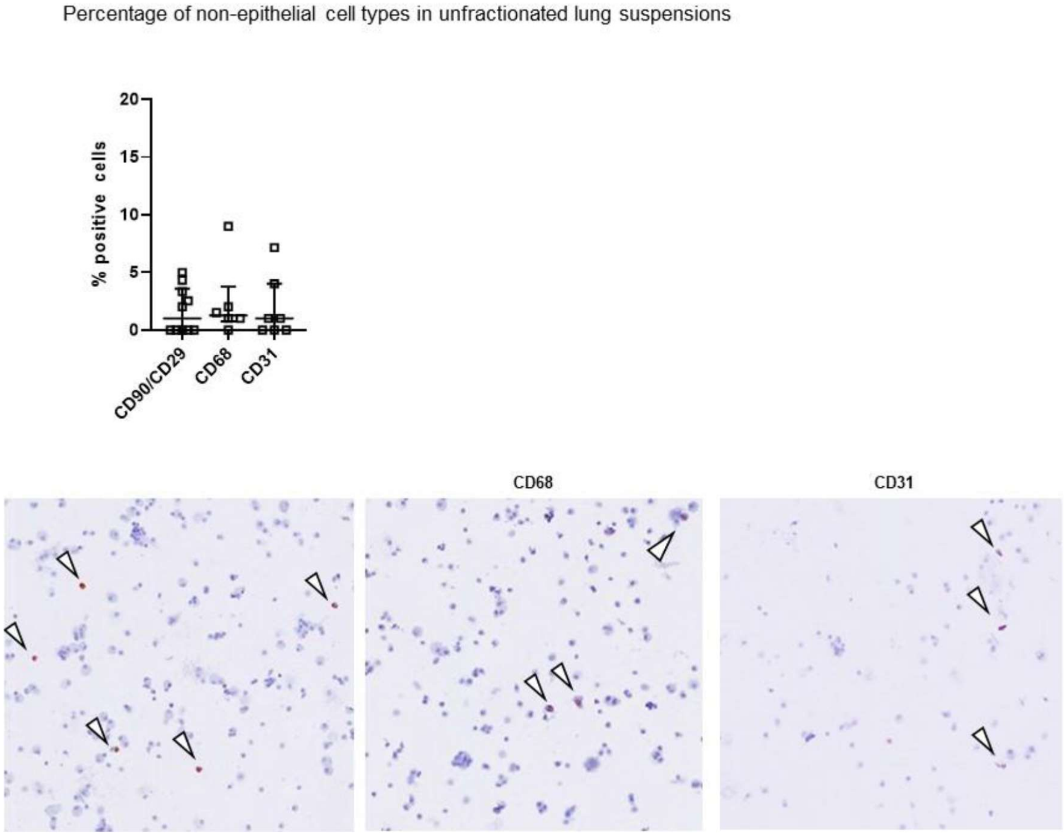

Figure S4. Percentage of additional cell types in the different cellular fractions used for organoid assays. Unfractionated cells suspensions were obtained by enzymatic digestion from human distal lung tissue. Cytospins were prepared from unfractionated suspensions were stained for CD90 or CD29 as stromal cell marker, CD68 as macrophage marker and CD31 as endothelial marker. Positive cells were quantified by counting of the complete cytospins by 2 independent observers and expressed as percentage. Medians are indicated and representative images are shown. Positive cells are indicated by arrows.
